# Adenosquamous Carcinoma of the Gallbladder: A Rare Surgical Entity

**DOI:** 10.7759/cureus.77811

**Published:** 2025-01-22

**Authors:** Anastasia D Karampa, Georgios Gkizas, Gerasimia Kyrochristou, Vasileios Tatsis, Vasileios Nousias, Dimitrios J Cyrochristos, Michail Mitsis, Angelos Liontos, Georgios D Lianos

**Affiliations:** 1 Department of Surgery, University Hospital of Ioannina, Ioannina, GRC; 2 Department of Internal Medicine, University Hospital of Ioannina, Ioannina, GRC

**Keywords:** adenosquamous carcinoma, anemia, cholecystostomy, gallbladder cancer, gastrointestinal bleeding, gastrojejunostomy

## Abstract

Gallbladder cancer (GBC), while infrequent, is the predominant malignancy of the biliary tract. The absence of clinical symptoms in conjunction with aggressive behavior contributes to delayed diagnosis and unfavorable prognosis. This condition typically remains asymptomatic in its initial stages, making detection challenging.

Herein, we report a case of adenosquamous carcinoma of the gallbladder in a 74-year-old Caucasian woman who was admitted due to anemia, gastrointestinal bleeding, and abdominal pain.

Computed tomography (CT) of the abdomen demonstrated a formation adjacent to the neck of the gallbladder. A lesion with unclear boundaries in the IVb liver segment was also observed. CT-guided percutaneous biopsies were conducted on the thickened wall of the gallbladder and the suspicious area of the liver. The histological report from liver biopsy indicated carcinoma of the gallbladder characterized by squamous differentiation.

Τhe case was discussed in an oncology board meeting, and the patient underwent exploratory laparotomy. Intraoperative observations revealed an invasive gallbladder neoplasm extending to the hepatoduodenal junction and the second part of the duodenum. The surgical team performed cholecystostomy and gastrojejunostomy with palliative intent. Gallbladder wall biopsies were obtained intraoperatively and histopathology results confirmed the initial diagnosis of adenosquamous carcinoma. The patient was referred to an oncology board meeting, and it was decided to be a combination of chemotherapy and chemoradiation according to the National Comprehensive Cancer Network (NCCN) guidelines.

## Introduction

Gallbladder cancer (GBC) is the predominant malignancy of the biliary tract, accounting for two-thirds of all biliary tract lesions, with an annual incidence of 6,500 cases in the United States [1,2]. Adenocarcinomas represent the predominant variant of gallbladder neoplasms, whereas adenosquamous and pure squamous cell gallbladder carcinomas account for less than 10% of gallbladder tumors [3]. Significant risk factors for GBC encompass chronic cholelithiasis, obesity, female gender, porcelain gallbladder, gallbladder adenomatous polyposis, *Salmonella typhi *infection, and abnormal pancreaticobiliary duct junction [4]. Although the incidence of GBC among patients with cholelithiasis is low (3%), gallstones are present in 70%-90% of individuals diagnosed with GBC [5]. Patients with GBC infrequently present with symptoms, and in the majority of instances, the diagnosis is established incidentally following an elective cholecystectomy for symptomatic cholelithiasis. Early detection remains challenging, as surgical resection is currently the sole treatment option with potential curative intent [1].

## Case presentation

A 74-year-old Caucasian woman was admitted to our hospital due to debility and epigastric pain of recent onset. Her vital signs were within normal range. Upon physical examination, her abdomen was pliable and devoid of pain, exhibiting normal peristaltic sounds. Rectal examination resulted negative for bleeding (empty rectum). The patient did not report any prior surgeries or a history of alcohol or tobacco consumption. She was treated for arterial hypertension, hypothyroidism, and hyperlipidemia on a chronic basis. In the past month, the patient indicated the use of anti-inflammatory medication for one week to address a small pericardial effusion identified in a cardiac ultrasound.

A comprehensive blood test was conducted upon her admission. The laboratory findings are displayed in Table 1. Additionally, the hepatitis panel and tumor markers (CEA, CA 19.9, βHCG, AFP, CA 15.3, CA 125) were within normal limits.

**Table 1:** Patient’s laboratory tests on admission

Variables	Reference rate	On admission
Hemoglobin (g/dl)	11-15	8
Hematocrit (%)	35-42	26
White blood count (per mm^3^)	4500-11000	9400
Platelet count (per mm^3^)	150000-450000	456000
Erythrocyte sedimentation rate (mm/h)	0-30	90
INR	0.8-1.2	1.34
D-dimers (μg/ml)	<0.5	1.04
Creatinine (mg/dl)	0.6-1.2	0.89
Urea (mg/dl)	11-54	25
Total bilirubin (mg/dl)	0.1-1	0.6
Direct bilirubin (mg/dl)	0.01-0.2	0.11
Aspartate transaminase (U/L)	10-35	15
Alanine transaminase (U/L)	10-35	11
Alkaline phosphatase (U/L)	30-125	72
Amylase (U/L)	0-90	30
Creatinine kinase (U/L)	25-160	62
Lactate dehydrogenase (U/L)	115-230	134
Hs-troponin (pg/ml)	0-11.6	3.9
Iron (μg/dl)	50-150	18
Ferritin (ng/ml)	11-306	138
Vitamin B12 (pg/ml)	145-914	340
Folic acid (ng/ml)	3.1-19.9	12.6

Initial endoscopy identified a friable region in the D2 segment of the duodenum with active bleeding. Hemostasis was performed on an emergency basis upon admission (secondary endoscopy), utilizing an adrenaline solution and hemostatic clips. Biopsies were obtained from the ulcerated area.

**Figure 1:**
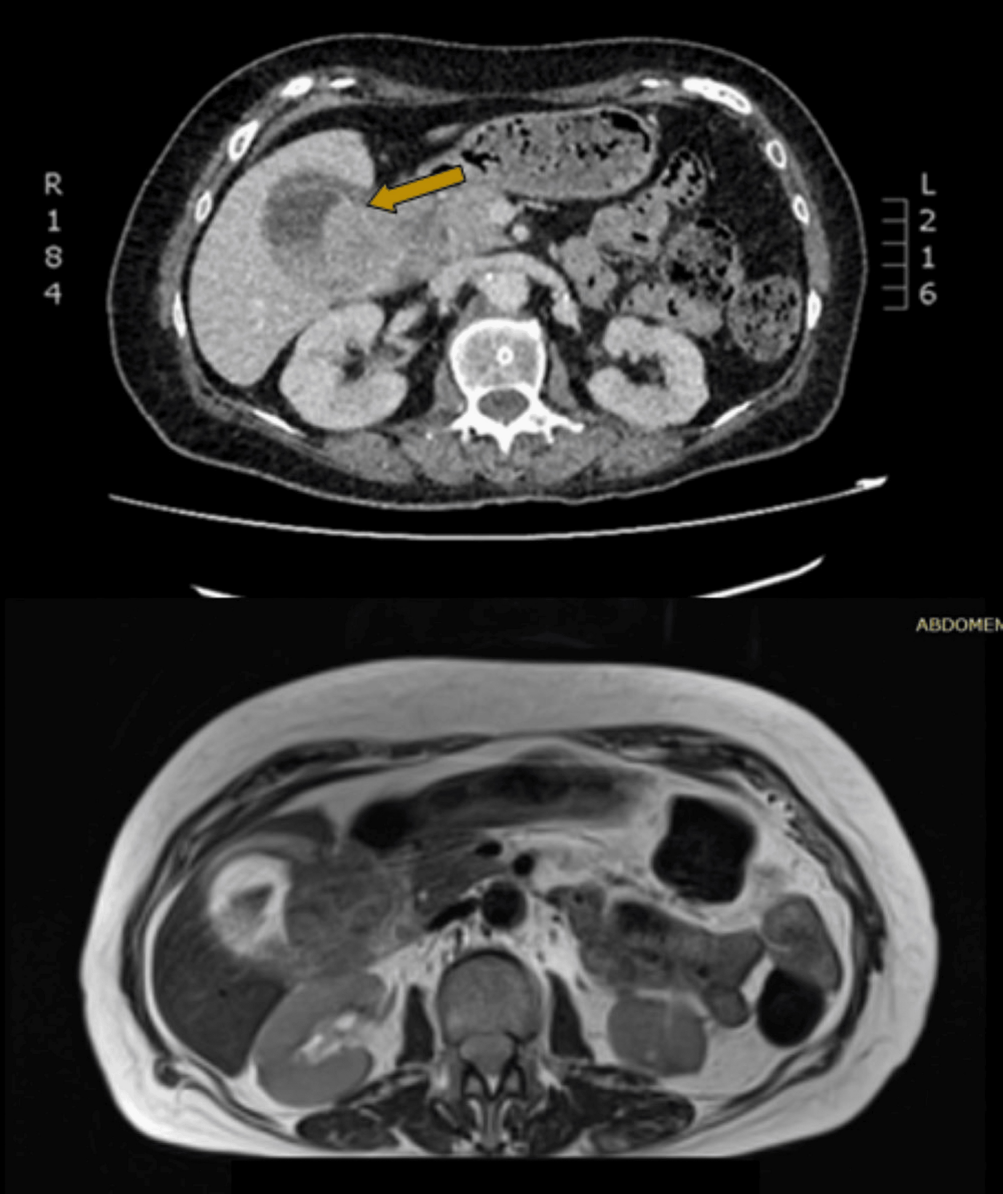
Coronal contrast-enhanced CT (top) and MRI T2-weighted (bottom) images of the abdomen illustrate a gallbladder mass lesion CT: computed tomography; MRI: magnetic resonance imaging

**Figure 2:**
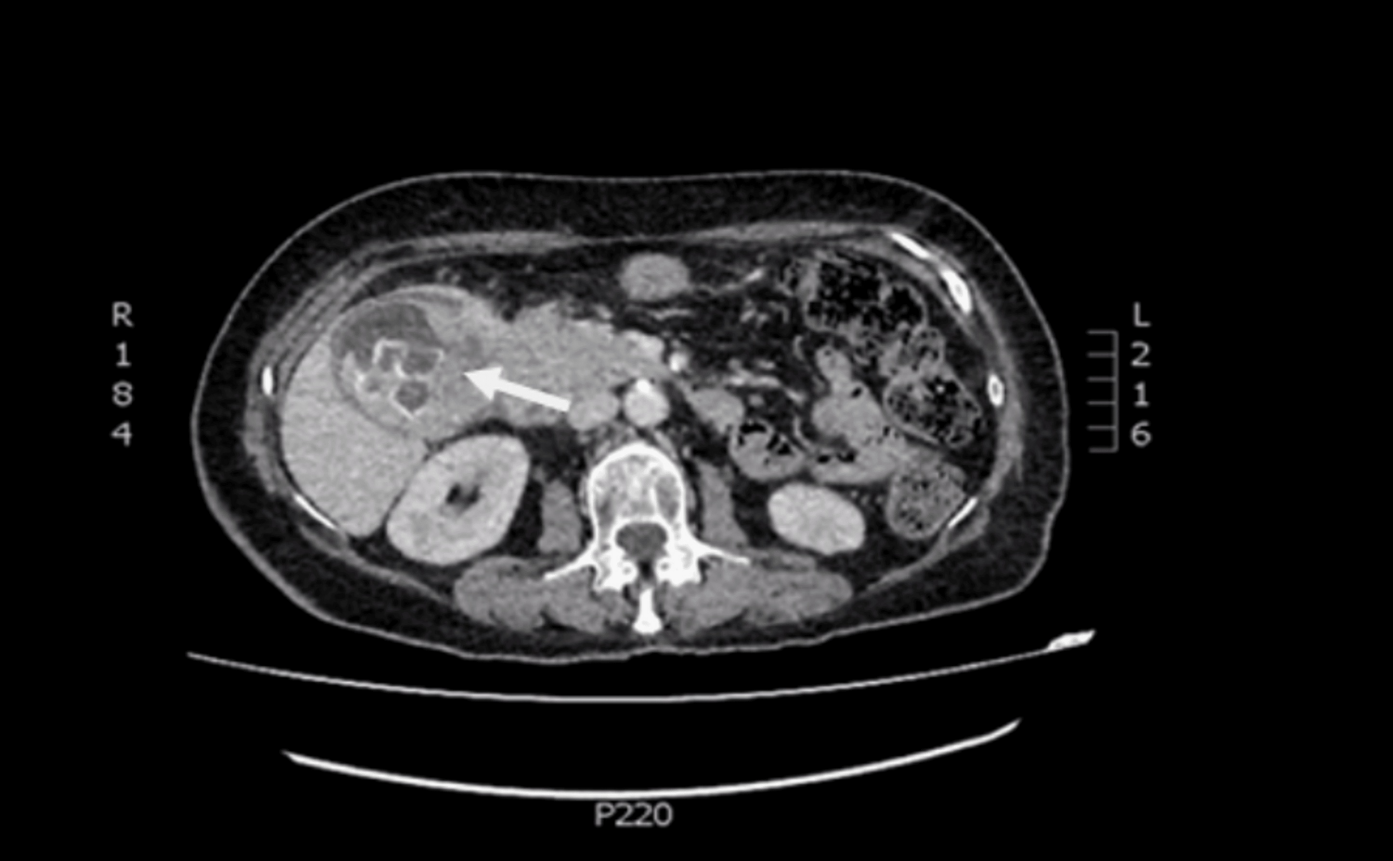
Coronal contrast-enhanced CT image of the abdomen demonstrates gallbladder mass lesion and gallbladder stones CT: computed tomography

**Figure 3:**
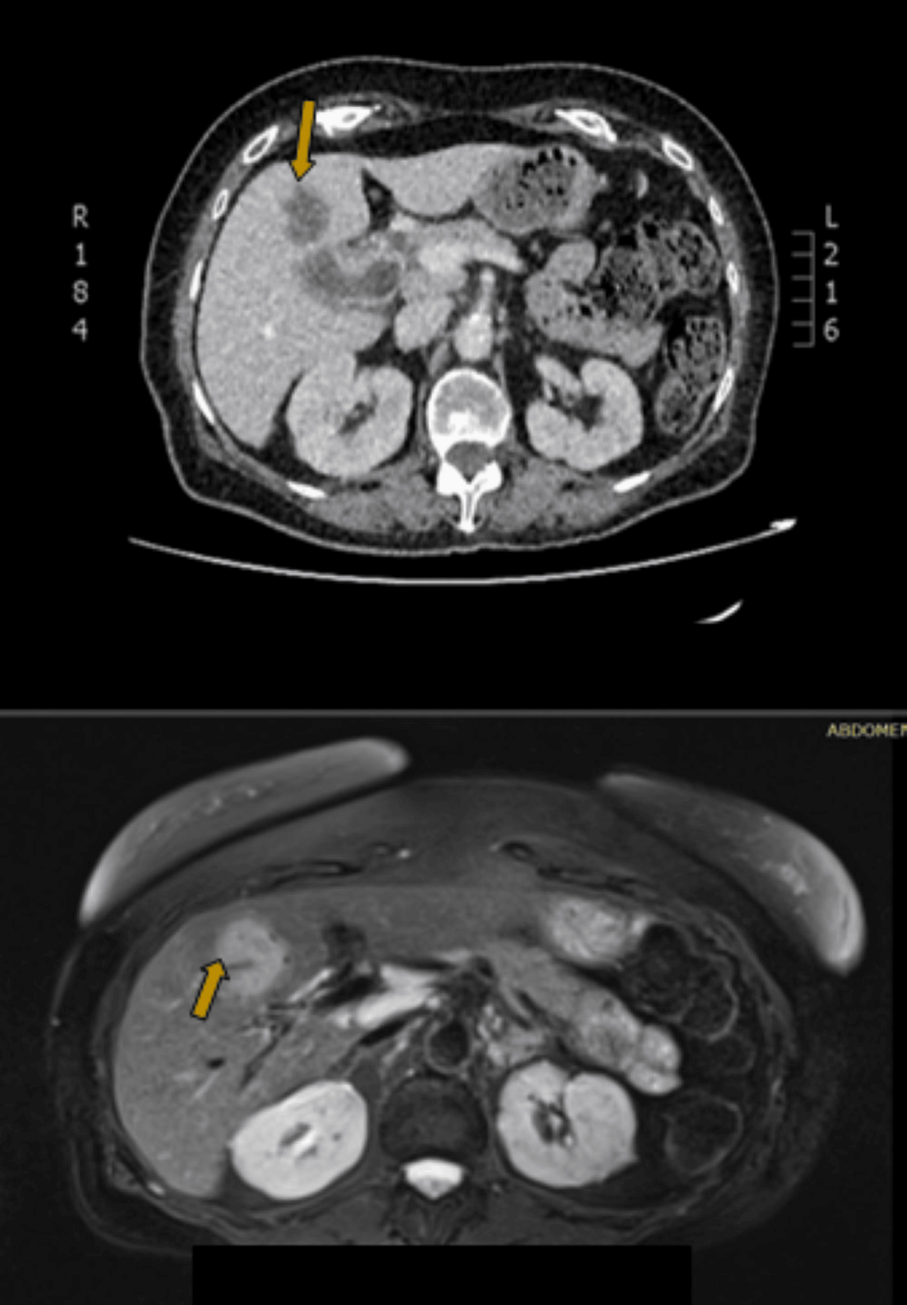
Coronal contrast-enhanced CT image (top) alongside an MRI T2-weighted image (bottom), illustrating gallbladder wall thickening and a heterogeneously enhanced lesion with unclear margins in the IVb liver segment.

Computed tomography (CT) of the abdomen demonstrated a 5.5 x 6.5 cm formation adjacent to the neck of the gallbladder, protruding from its wall and extending intraluminally with notable contrast enhancement (Figures 1a-1b, 2). Additionally, there were found enlarged regional lymph nodes and a loss of the normal fat layer between the gallbladder wall and the pylorus, as well as the D1 and D2 portions of the duodenum. A heterogeneously enriched lesion with unclear boundaries in the IVb liver segment was also observed (Figures 3a-3b).

CT-guided percutaneous biopsies were conducted on the thickened wall of the gallbladder and the suspicious area of the liver in order to exclude the possibility of liver abscess, given that tumor markers were within normal limits as mentioned before. The histological report from liver biopsy indicated carcinoma of the gallbladder characterized by squamous differentiation, comprising both squamous and glandular components. According to the histopathology report, cancer has grown through the serosa, and it has grown directly into the liver (stage T3). The most probable diagnosis was "adenosquamous carcinoma of the gallbladder."

**Figure 4:**
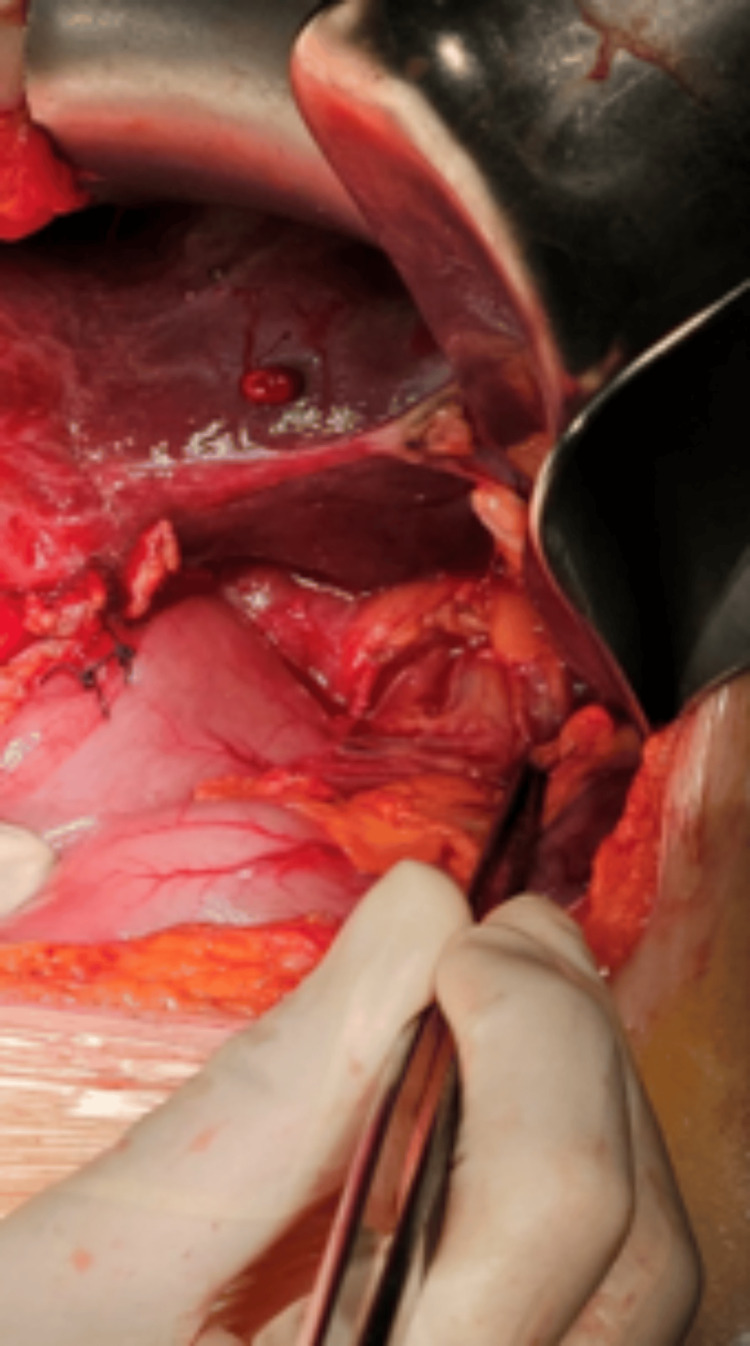
Intraoperative picture. Hepatoduodenal junction infiltration

**Figure 5:**
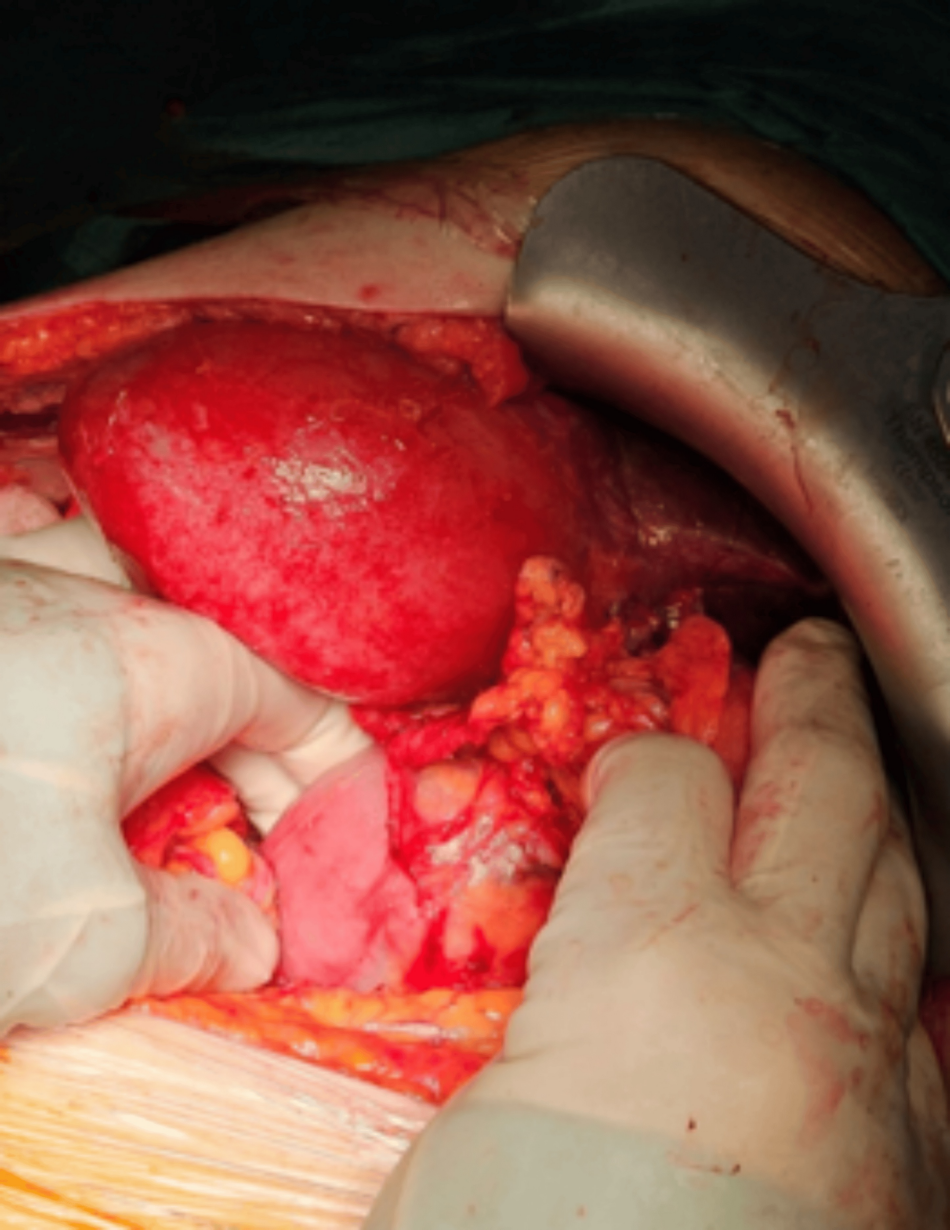
Intraoperative picture. Invasive gallbladder neoplasm with sufficient wall thickening. Extension to the D2-part of the duodenum is observed

A comprehensive review of the patient's case occurred at the multidisciplinary tumor oncology board of our hospital. Given the patient's previous good performance status and the lack of distant metastatic lesions, surgical intervention was determined to be appropriate. The chosen surgical approach was exploratory laparotomy performed under general anesthesia via a midline incision. Intraoperative observations revealed an invasive gallbladder neoplasm extending to the hepatoduodenal junction (Figure 4) and the second part of the duodenum (Figure 5). The surgical team performed cholecystostomy and gastrojejunostomy with palliative intent, in order to avoid symptoms of obstructive jaundice. Spread to nearby lymph nodes could not be excluded, since enlarged regional lymph nodes were found at the hepatoduodenal ligament. However, no lymph node biopsy was performed to confirm the diagnosis. The patient exerted a successful postoperative recovery and was discharged on the eighth postoperative day. Gallbladder fundal wall biopsies were obtained intraoperatively and histopathology results confirmed the initial diagnosis of adenosquamous carcinoma. The patient was subsequently referred to the oncology board meeting, and a combination of chemotherapy (gemcitabine and cisplatin) and chemoradiation was decided according to the National Comprehensive Cancer Network (NCCN) guidelines.

## Discussion

GBC is of rare occurrence among gastrointestinal malignancies. It is associated with poor prognosis, due to nonspecific clinical manifestations and delayed diagnosis. The age of onset ranges from 43 to 89 years old (average 66.6 years) [4], and there is a clear predominance for females compared to men (ratio 3:1). The global prevalence ranges from 0.7 to 21 per 100,000, while in the United States, it is approximately 1 to 2 per 100,000. Histological subtypes of GBC include adenocarcinoma (90%), with less frequent occurrences of adenosquamous carcinoma (5%-10%), squamous cell carcinoma (1%-6%), and oat cell carcinoma. Adenosquamous cell carcinoma comprises both glandular and squamous components. The five-year survival rate is below 5%, with an average survival duration of less than six months. Clinical symptoms may include right upper quadrant abdominal pain (with or without a palpable liver mass), rapid satiety, anorexia, weight loss, weakness, nausea, and vomiting, often indicating inoperable disease [6].

Recent literature indicates that patients with gallbladder pathologies, when malignancy is suspected, should be referred for abdomen-pelvis CT in conjunction with magnetic resonance imaging (MRI), utilizing cross-sectional and arterial phase images for staging and assessment of resectability. The tumor, node, metastasis (TNM) staging system, developed by the American Joint Committee on Cancer (AJCC) and the Union for International Cancer Control (UICC), is the predominant classification framework. The enhanced proliferative capacity of squamous components contributes to an elevated T stage [4,6].

Treatment modalities are scarce due to the aggressive biological behavior of this enigmatic clinical entity. At stages I, IIA, and IIB where the tumor is localized within the gallbladder and does not involve adjacent structures or locoregional lymph nodes, curative surgical resection via cholecystectomy remains the primary treatment approach, achieving a five-year survival rate of 100%. Cholecystectomy, in conjunction with central hepatectomy and regional lymphadenectomy, followed by chemotherapy (either systemic or regional), is indicated for early-stage lesions exhibiting liver invasion. Locally advanced disease (stages IIIA, IIIB) necessitates treatment through extended radical resection, which may encompass extended right hemihepatectomy, vascular reconstruction, extended lymphadenectomy, extrahepatic bile duct resection, and, when indicated, combined pancreaticoduodenectomy [2]. Chemotherapy, targeted therapy/immunotherapy, and chemoradiation are utilized in advanced-stage patients where surgical resection with no cancer cells seen microscopically at the primary tumor site (R0) margins is not possible, or to enhance prognosis for those with positive resection margins and/or infiltrated lymph nodes [7,8]. Recent evidence indicates that the combination of gemcitabine and cisplatin is the most effective treatment for advanced inoperable or metastatic disease [2,6].

## Conclusions

GBC is a unique condition characterized by elevated mortality rates. Patients frequently exhibit enlarged tumors that involve adjacent organs, rendering them typically inoperable. Despite advancements in treatment options, the prognosis remains significantly unfavorable, attributable to high invasiveness and tumor heterogeneity. The primary objective for gallbladder adenosquamous carcinoma is to achieve early diagnosis at a stage amenable to curative intervention, facilitating radical surgical excision with therapeutic intent. Unfortunately, in the case of our patient, the symptomatology was unclear, and the patient sought medical assistance at an advanced stage of the disease. At present, there are no established treatment guidelines for this rare and aggressive cancer type. Next-generation sequencing (NGS) for mutational profiling may aid clinicians in identifying and treating actionable mutations in this rare neoplastic disease by elucidating the pathogenesis of GBC and the mechanisms linked to inflammation, tumor progression, invasion, and metastasis. According to the NCCN guidelines, treatment for GBC includes surgery (stages I, IIa, IIB, IIIA), chemotherapy, targeted cancer drugs, and radiotherapy (IIIB, IV). Further studies are essential to establish a more targeted and personalized treatment approach to improve outcomes for these patients.
